# Abundance of *Lutzomyia longipalpis* in urban households as risk factor of transmission of visceral leishmaniasis

**DOI:** 10.1590/0074-02760150366

**Published:** 2016-05

**Authors:** Elisa Neves Vianna, Maria Helena Franco Morais, Andréa Sobral de Almeida, Paulo Chagastelles Sabroza, Ilka Afonso Reis, Edelberto Santos Dias, Mariângela Carneiro

**Affiliations:** 1 Universidade Federal de Minas Gerais, Faculdade de Medicina, Pós-graduação em Ciências da Saúde, Infectologia e Medicina Tropical, Belo Horizonte, MG, Brasil; 2 Prefeitura de Belo Horizonte, Secretaria Municipal de Saúde, Belo Horizonte, MG, Brasil; 3 Fundação Oswaldo Cruz, Escola de Saúde Pública, Departamento de Doenças Endêmicas, Rio de Janeiro, RJ, Brasil; 4 Universidade Federal de Minas Gerais, Instituto de Ciências Exatas, Departamento de Estatística, Belo Horizonte, MG, Brasil; 5 Fundação Oswaldo Cruz, Centro de Pesquisas René Rachou, Laboratório de Leishmanioses, Belo Horizonte, MG, Brasil; 6 Universidade Federal de Minas Gerais, Instituto de Ciências Biológicas, Departamento de Parasitologia, Belo Horizonte, MG, Brasil

**Keywords:** Lutzomyia longipalpis, household characteristics, visceral leishmaniasis

## Abstract

Urban occurrence of human and canine visceral leishmaniasis (VL) is linked to households with characteristics conducive to the presence of sand flies. This study proposes an *ad hoc* classification of households according to the environmental characteristics of receptivity to phlebotominae and an entomological study to validate the proposal. Here we describe the phlebotominae population found in intra- and peridomiciliary environments and analyse the spatiotemporal distribution of the VL vector *Lutzomyia longipalpis* of households receptive to VL. In the region, 153 households were classified into levels of receptivity to VL followed by entomological surveys in 40 of those properties. Kruskal-Wallis verified the relationship between the households’ classification and sand fly abundance and Kernel analysis evaluated *L. longipalpis* spatial distribution: of the 740 sand flies were captured, 91% were *L. longipalpis*; 82% were found peridomiciliary whilst the remaining 18% were found intradomiciliary. No statistically significant association was found between sandflies and households levels. *L. longipalpis* counts were concentrated in areas of high vulnerability and some specific households were responsible for the persistence of the infestation. *L. longipalpis* prevails over other sand fly species for urban VL transmission. The entomological study may help target the surveillance and vector control strategies to domiciles initiating and/or maintaining VL outbreaks.

Visceral leishmaniasis (VL) is a neglected tropical disease with an incidence between 200,000 and 400,000 new cases and over 20,000 deaths occurring worldwide each year (WHO 2015). In the Americas, the disease is caused by the protozoan *Leishmania (Leishmania) infantum* and its main vector is *Lutzomyia (Lutzomyia) longipalpis* (Lainson & Rangel 2005, WHO 2015).

In Brazil, *L. longipalpis* is found not only in forests but also in urban environments (Lainson & Rangel 2005, Fernández et al. 2010), particularly in areas with some vegetation (de Oliveira et al. 2012, Fernández et al. 2013). Other species such as *Lutzomyia cruzi* and *Lutzomyia evansi* are restricted to the Brazilian state of Mato Grosso do Sul and Colombia, respectively (Travi et al. 1990, Santos et al. 1998, 20, 2003). These species transmit *L. infantum* to humans and other mammals including canines and rodents (Gontijo & Melo 2004).

Between 2011 and 2013, over 3,000 cases of VL were reported in Brazil with a fatality rate of 6.7% in 2013 (PAHO 2015). The city of Belo Horizonte, capital of the Brazilian state of Minas Gerais (MG), has recorded cases of human VL since 1994 (Silva et al. 2001). The incidence of human VL in Belo Horizonte for the period between 2006 and 2014 increased from 1.6 to 5.5 cases per 100,000 inhabitants whilst the fatality rate ranged between 12.8 and 21.4%. In the same period, the canine seroreactivity to *L. infantum* increased from 3.2 to 13.9% (PBH 2015). The presence of the vector *L. longipalpis* was recorded in the city in 1978 in parks forests (Martins et al. 1978) and between 1997-2014 at high and low densities in different areas of the municipality both intra- and peridomiciliary (Souza et al. 2004, Resende et al. 2006, Saraiva et al. 2011, Lara-Silva et al. 2015).

Poor living conditions and poor sanitation are associated with human and canine VL infections by *L. infantum* in urban areas (Moreira et al. 2003, Moreno et al. 2005, Oliveira et al. 2006b, Coura-Vital et al. 2013). Moreover, areas with homes surrounded by trees with large canopies or vegetation (de Oliveira et al. 2012, Fernández et al. 2013), with dumping of disposable material in the soil (Fernández et al. 2013) and hens in peridomicile (Quintana et al. 2012) favor infections by enabling the survival and reproduction of the vector and facilitates transmission in the urban and periurban environments. Thus, the identification of households presenting characteristics that favors the colonisation and proliferation of the vector and, thereby, the transmission of VL (i.e. vulnerability) can help vector control strategies in urban areas.

The Brazilian Visceral Leishmaniasis Surveillance and Control Program (VL-SCP) aims to reduce the risk of transmission as well as the mortality rates and the degree of morbidity of this disease through rapid diagnosis and early treatment of human cases. To this end, the Control Program has adopted reservoir and vector control strategies targeted to high risk areas focusing on health education and environmental management (MS 2006). However, these strategies are frequently not enough to curb the spread of the disease, particularly in big cities. Thus, the development of a methodology to categorize households according to their receptivity to the spread of VL may provide insightful information to optimise the VL-SCP resources to control vector dispersion and VL transmission in urban areas.

This study proposes an *ad hoc* classification of households according to the environmental characteristics of receptivity to phlebotominae and an entomological study to validate the proposal. Its analyses the diversity of sand flies according to the level of receptivity of households classified by health agents of Brazilian system of public health. It reveals the spatiotemporal distribution of *L. longipalpis* in households identified as receptive to the transmission of VL in a neighborhood of the northwestern region of Belo Horizonte, Brazil.

## MATERIALS AND METHODS

*Study area* - Belo Horizonte is a city of 2,491,109 inhabitants in 331,401 Km^2^, a population density of 7,167 inhabitants/Km^2^ (IBGE 2015). The climate fits the Aw class, which means a tropical climate (A) with summer rains (w) and average altitude of 852 m, according to the Köppen classification (Köppen & Geiger 1928).

The study was conducted by the Health Department of the Northwestern District of Belo Horizonte, which is assisted by the Pindorama Health Centre (PHC) covering a population of 20,672 inhabitants and a population density of 12,920 inhabitants/Km^2^. According to the Zoonosis Control Unit of the Northwest Health District, the PHC coverage area had 2,415 dogs in 2012 and 2,423 in 2013. The northwest region of Belo Horizonte was selected because of the high coverage and good continuity of the VL control activities over the past few years (2006-2013), which included canine census and vector control strategies targeted to annually prioritised micro regions. The LVH (human LV) incidence rate to the region in 2006 to 2014 ranged from 31.4-5.2 and canine seroreactivity for *L. infantum* from 7.3-4% (PBH 2015).

**Fig. 1:**
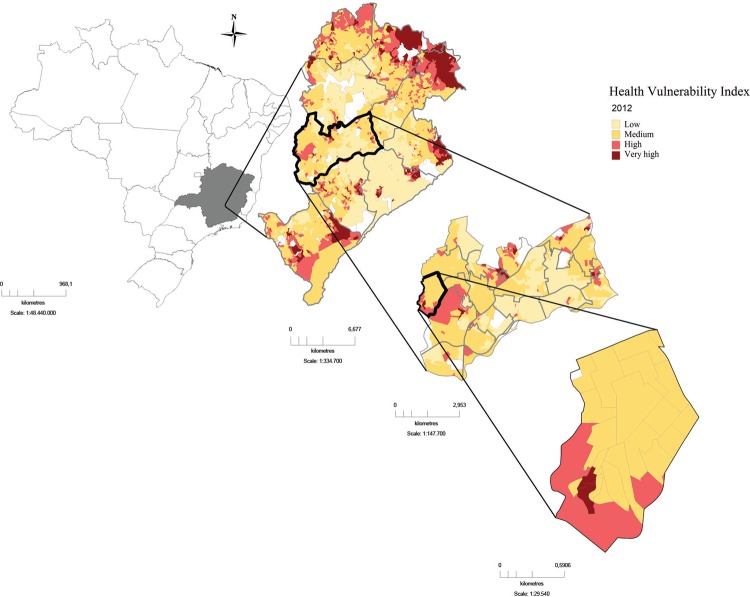
: Vulnerability Index map of Belo Horizonte, Minas Gerais, Brazil, and study area (northwestern region).

The region studied includes areas of high indexes of risk to health according to the Health Vulnerability Index (HVI) developed by the Health Department of Belo Horizonte. The HVI of 2012 was determined for each of the 2,563 census sectors of the city according to the following indicators: sanitation, housing, income, education and health (HVI 2015). This index allows the classification of the census sectors into four health vulnerability categories: low, medium, high and very high (Fig. 1). In some districts, the VL control measurements are active only in the microareas where the HVI is high. In Belo Horizonte, the HVI is commonly used in health policy and planning. Furthermore, some indicators such as income and education were associated with the risk of becoming ill by VL (de Araújo et al. 2013).

*Sample calculation* - The study area comprises 9,233 households. From these, the Health agents from the Brazilian public health selected a total of 153 by convenience sampling, according to the presence of characteristics, raised in the literature. Selected houses had a history of presence of positive dogs for LV at domicile or in the neighborhood. This sample included households classified to a four differentiated levels of receptive to the risk of VL infection (including control level) according to physical and environmental characteristics of intradomiciliary and peridomiciliary contexts. The criteria for these ratings are shown in Table I. A random subsample of 40 households was selected for entomological assessment.

**TABLE I:** Criteria used by Endemy Control Agents and field supervisors from Brazilian system public health to classify the household risk of Visceral leishmaniasis transmission and abundance of sand flies

Criteria for the classification of receptive/vulnerable households	Level
Positive canine and/or human case for *Leishmania infantum* at the domicile or in the neighboring home	3
Presence of a lot of organic matter, fruits, animal feces on the ground of the peridomicile	
Presence of trees and shrubs in the peridomicile	
Presence of more than one animal species Shaded peridomicile (tree canopies) Presence of decomposing organic matter on the ground	
Presence of holes and/or cracks (on the wall, ceiling), presence of attic	
	
Positive canine and/or human case for *L. infantum* at the domicile or in the neighboring home	2
Presence of organic matter on the ground	
Presence of trees and shrubs in the peridomicile	
Presence of one animal species	
Walls and ceiling in good structural conditions	
	
Positive canine and / or human case for *L. infantum* at the domicile or in the neighboring home	1
Presence of little organic matter on the ground	
No animal husbandry	
Walls and ceiling in good structural conditions	
	
Positive canine and / or human case for *L. infantum* at the domicile or in the neighboring home	0
Partially or fully cemented yard Absence of any of the above listed characteristics in the peridomicile and intradomicile	

The receptive households were defined as those presenting conditions conducive to the colonisation and proliferation of sand flies such as shading, soil organic matter, vegetation, soil moisture and presence of animals. These households were selected for sand fly surveys in urban areas because they have environmental characteristics associated with a high risk of VL infection in urban areas (Lainson & Rangel 2005, Moreno et al. 2005, Oliveira et al. 2006b, Resende et al. 2006, Fernández et al. 2010, 20, 2013). Buildings classified at level three were receptive and considered vulnerable to sand flies invasion, according to the condition of the walls, windows and roofs such as the presence of holes and cracks or the absence of plaster. Indeed, unplastered adobe buildings have been associated with the risk of infection by *L. infantum* in Belo Horizonte (Coura-Vital 2013). Other features considered were the presence of at least one canine and/or human case in the residence or in the vicinity over the past five years.

*Entomological surveys* - The sampled households were surveyed every two months for three consecutive nights, from May 2012-June 2013, totaling seven periods of capture. Two HP light traps (Pugedo et al. 2005) were used: one inside the house and another in the peridomicile. Traps were placed approximately 1.5 m above the ground as recommended by the VL-SCP manual (de Araújo et al. 2013). Traps were installed in places considered more likely to attract sand flies such as fruit trees, balconies, henneries, barnyards, gardens and in the walls surrounding the peridomiciles. The traps sampled the sites, intra e peridomicile in 40 domiciles, 1680 times totaling 13.440 hours of capture.

The sand fly specimens were first identified using a magnifying glass, and prepared, clarified and mounted as described in the Langeron protocol (Langeron 1949). The specimens’ subgenus, genus and species were identified using optical microscopy according to Young and Duncan (1994).

To verify the association between entomological data and households classification for receptivity was performed the test of Kruskal-Wallis. The explanatory variable was “receptivity level/of households” and the outcome variables were considered: “total number of sand flies captured in intradomicile”; “total number of sand flies captured in peridomicile” and “total number of sand flies in domicile”. Significance level of 5% was considered in the test.

*Spatial statistics analysis* - The 40 households selected from the neighborhood of the PHC were geo-referenced by their addresses using a global positioning system (GPS) receiver and referenced into the Universal Transverse Mercator (UTM) projection. Subsequently, we analysed the intra- and peridomiciliary population densities, from the abundance of the LV vector *L. longipalpis* in each of the seven periods studied, using the kernel estimate. Maps of vector densities were designed to analyse hotspots in space and time using the quartic kernel function. The degree of smoothing was controlled by a bandwidth with a radius of 200m. This analysis reveals the intensity of a given characteristic, in this study the *L. longipalpis* counts captured in the intra- and peridomicile areas.

The kernel density estimate is a suitable interpolation technique for the positions of individual points based on a mathematical model that creates a symmetrical surface on each point, by assessing the distance of the point to a reference position and then adding the value of all such surfaces to the reference position (Bailey & Gatrell 1995, Gatrell et al. 1996).

## RESULTS

Seven hundred and forty sand flies were captured in the 40 selected households. However, only 733 specimens were actually considered because, upon withdrawal of the traps, seven suffered anatomical damages that compromised the identification of their domicile of origin. The Kruskal-Wallis test revealed no difference in household levels and the sand flies abundance in intra-, peri- and domicile. In spite of the results, it was observed difference between sand flies counts in the domiciles; the difference was not enough to identify the most receptive properties according to the characteristics studied (Table II).

**TABLE II:** Classification of households according to the level of receptivity and vulnerability, rate of infested households, and total counts and median of sand flies captured in the sampled buildings

Receptivity level	Households	Infested households (%)	Sand flies (N/Median)		
0	4	3 (75)	2/0,5	16/2	18/2,5
1	7	5 (71.4)	3/0	141/1	144/1
2	11	10 (90.9)	43/2	300/1	343/3
3	18	14 (77.7)	95/1	133/1,5	228/4
Total	40	32 (85)	143	590	733

91% of the captured sand flies were identified as *L. longipalpis* (670 specimens), 4.8% were *Lutzomyia cortelezzii*, 0.27% were *Lutzomyia whitmani,* and 0.14% were *Lutzomyia longispina.* Due to anatomical damages inflicted upon withdrawal of the traps, 4% of the captured sand flies were identified only in terms of genera. As shown in Table III, 20 males and 16 females were identified as *L. cortelezzii* (Galati et al. 1989). The ratio of males to females was 7:3. The highest numbers of captured sand flies were in January and April 2013 (Table III).

**TABLE III:** Sand fly species captured in the sampled households northwest regional, Belo Horizonte, Minas Gerais, Brazil, from May 2012-June 2013

Month/Year	*Lutzomyia longipalpis*		*Lutzomyia cortelezzii*		*Lutzomyia whitmanni*		*Lutzomyia longispina*		*Lutzomyia* sp.	Total
	F	M	F	M	F	M	F	M		
May/2012	11	73	0	4	1	0	0	0	9	98
July/2012	4	13	0	1	0	0	0	0	0	18
September/2012	6	37	0	2	0	0	0	0	3	48
November/2012	20	75	3	3	0	1	0	0	0	102
January/2013	4	99	3	4	0	0	0	0	5	115
April/2013	29	221	10	5	0	0	0	1	12	278
June/2013	1	77	0	1	0	0	0	0	2	81
Total	75	595	16	20	1	1	0	1	31	740*

F = female; M = male; *Seven specimens could not be properly ascribed to their domicile of origin.

**Fig. 2:**
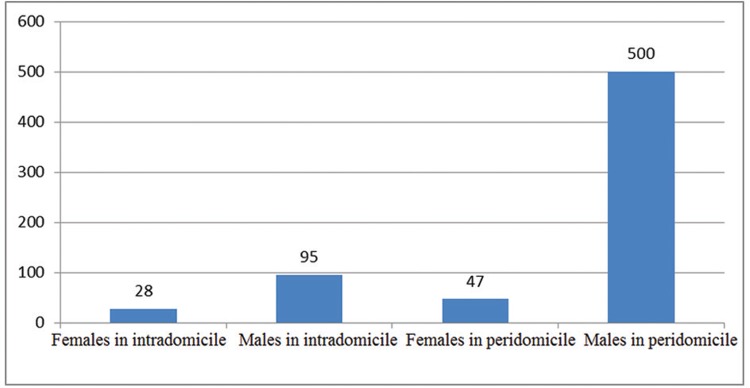
: number of *Lutzomyia longipalpis* captured in the peri- and intradomicile areas, separated by gender, demonstrating the exophilic and endophilic behaviors of the populations. Northwestern region of Belo Horizonte, Minas Gerais, Brazil.

Of the captured sand flies identified as *L. longipalpis*, 82% (547) were found in the peridomicile and 18% in the intradomicile areas (Fig. 2). *L. longipalpis* was 1.1 in the peridomicile and 3.4 in the intradomicile.

**Fig. 3:**
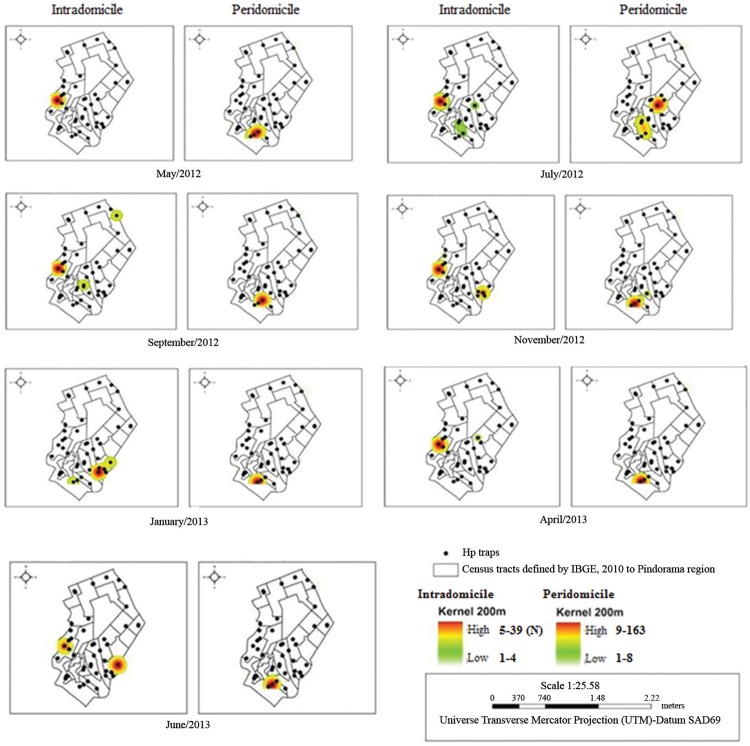
: distribution of properties sampled in the area studied and the abundance of *Lutzomyia longipalpis* in the peri- and intradomicile areas according to kernel analysis. Northwestern region of Belo Horizonte, Minas Gerais, Brazil.

The spatiotemporal analysis by kernel estimation revealed that the density of *L. longipalpis* was in the households located in the central, north, especially in south regions of the area studied. Shades of red and orange depict regions of the map presenting greater density and abundance of sand flies, whereas shades of yellow and greens represent regions with lower densities. Some buildings were identified as hotspots for the abundance of *L. longipalpis* collected intra and peridomiciliarily, which oscillated along each capture period (Fig. 3). The region with the highest number of continually infested buildings was located to the south of the area studied, where settlements of low socioeconomic status are found (areas with high vulnerability index).

## DISCUSSION

This study revealed the infestation profile of households by sand flies in the northwestern region of Belo Horizonte subjected to a systematic intervention of the VL-SCP. It proposes a system to classify the households as receptive to infestation by sand flies that revealed no difference in the number of captured sand flies in the domiciles classified in levels of receptivity. These results showed the difficulties to identify households that present fixed and standardised characteristics of degrees of receptivity levels. Moreover, the captures in intra- and peridomicile showed a pattern of spatiotemporal aggregation of *L. longipalpis* populations in specific areas from the surveyed households.

The area surveyed showed households with environmental differences often related to socioeconomic conditions, such as receptive homes level three, which were considered vulnerable to vector invasion. The level one houses, which apparently had not receptive conditions, were triggered by the houses of the neighborhood, which showed higher levels. The environmental conditions of peridomestic in the region are very similar, occurring low macrohabitats detection (considering intra- and peridomicile) using entomological data.

Factors associated with the risk of human VL infection include the presence of dogs (infected or not), the proximity to areas of green coverage, poor sanitary and housing conditions and the population density of the vector *L. longipalpis* (Quinnell & Dye 1994, Oliveira et al. 2001, Margonari et al. 2006, d, de Araújo et al. 2013). Receptive households are strategic locations to install sand fly traps in urban areas (Lainson & Rangel 2005, Resende et al. 2006, Santini et al. 2012, Fernández et al. 2013). Most of the households level two and three had large yards with the characteristics listed in Table I. The such as the presence of soil organic matter (Feliciangeli 2004) at least one animal specie (maintained at high density or not), the presence of trees and shrubs outside the home and intradomiciles in poor structural condition (presence of attic, broken windows, holes and cracks). These peridomicile areas have sites that favor the breeding of the sand fly larvae such as logs, hollow trees, roots and soil with faeces (Feliciangeli 2004, Sangiorgi et al. 2012). These large peridomicile areas of the PHC neighborhood classified in these levels may be responsible for the maintenance of potential breeding and/or sand fly outbreaks in the region.

The vector densities varied along time, with outbreaks appearing in one location one month and somewhere else at another point in time. This observation suggests a dynamic variation in the *L. longipalpis* abundance distribution throughout the year in the sampled houses, as proposed by Fernández et al. (2013). The present study identified areas of low and high vector aggregation. Salomón et al. (2004) suggested that the distribution of *L. longipalpis* is heterogeneous and the “source populations” are distributed into a geo-spatial pattern of high and low abundance regions. Using the kernel estimate, Almeida et al. (2011) found high-risk areas for human VL were concentrated in the outskirts of Teresina, the capital city of the Brazilian northeastern state of Piauí, which followed a heterogeneous distribution pattern over the period analysed. It is therefore plausible to think possible that the population of *L. longipalpis* population would follow a similar distribution pattern, perhaps even for other big cities of Brazil. Indeed, Camargo-Neves et al. (2001) evaluated the distribution of vector density in the city of Araçatuba in the southeastern Brazilian state of São Paulo and found a heterogeneous distribution of the vector, similar to that shown by the maps derived from the kernel analysis presented here. These studies support our findings suggesting a focal pattern of spatial infestation of sand flies in the households.

The households with abundant and persistent infestation over the study revealed different characteristics as to the other with respect to the trapped animals like chickens and dogs in dark and humid places, the presence of organic matter in the ground, shading in peri- and intradomicile, and presence of leafy trees, (levels two and three). The presence of different animal species at peridomicile may increase sand flies abundance, due to increased availability of hosts. *L. longipalpis* in rural areas has been captured near the horses, chickens, in burrows of armadillos and cavies (Ximenes et al. 1999).

A receptive household in LV transmission risk area should be prioritised, for example, for the environmental management. Find properties with persistent infestation over the months is important because and its may function as a supporting source of vectors throughout the year to areas around, hindering the natural environmental control due to climate conditions and rainfall. Persistence of *L. longipalpis* in the dry season months like autumn (April) probably indicates one (or more) breeding site being kept at home or around in peridomicile or neighborhood.

The effective control of VL in Brazil remains incipient because of numerous factors including the existence of gaps in the information on the dispersion and the location(s) of the vector(s) *L. longipalpis* and *L. cruzi*, the two main Brazilian VL vectors (Santos et al. 2003, Lainson & Rangel 2005, Pita-Pereira et al. 2008, Carvalho et al. 2010). The present study shows that *L. longipalpis* is still the main vector of VL in Belo Horizonte because it is the most commonly found species in the city´s residential areas, have been found naturally infected by *L. infantum* in the city (Saraiva et al. 2011, Lara-Silva et al. 2015). The results reported herein are in accordance to findings reported elsewhere (Souza et al. 2004, Resende et al. 2006, Lara-Silva et al. 2015).

A retrospective analysis of *L. longipalpis* infestation in Belo Horizonte and surrounding areas seems to reveal changes on this vector’s infestation pattern, which suggest an evolution in the process of urbanisation of this species similar to that reported for the city of Campo Grande, capital of the central western Brazilian state of Mato Grosso do Sul (Oliveira et al. 2006a). *Lut- zomyia longipalpis* presence in the metropolitan region of Belo Horizonte was first reported in 1978 and after to the early 1990s (Martins et al. 1978, Passos et al. 1993). Then, between 1997-1999, Resende et al. (2006) found that 39% of the sand fly specimens captured in the metropolitan area were *L. longipalpis* and, in the period between 2001-2003. Souza et al. (2004) reported this species represented 68.2% of the sand flies found in the city. More recently, Saraiva et al. (2011) and Lara-Silva et al. (2015) reported that *L. longipalpis* constituted 94% and 96.5% in the urban area of Belo Horizonte, respectively. Their data were similar to those revealed in the present study. These studies have used different sampling methods (CDC and Shannon traps) in diverse locations (green urban spaces or transition areas that do not include the peridomicile). Taken together, these observations indicate that this particular vector species stood out amongst the others over the last two decades. They confirm the invasive nature, ability to adapt to new ecological niches, opportunism and synanthropy of the most important VL vector species in the Americas (Rangel & Vilela 2008).

Among the other sand fly species captured in this study, only *L. whitmani* is a vector for human cutaneous leishmaniasis (Mayrink et al. 1979, Rangel & Lainson 2003). The other two species identified, *L. cortelezzii* and *L. longispina,* are not considered vectors of leishmaniasis. Although samples from *L. cortelezzi* have tested positive for *L. infantum* (Lana et al. 2015, Lara-Silva et al. 2015) or *Leishmania braziliensis* (Rosa et al. 2012, Lana et al. 2015) this species remains considered of no epidemiological importance in the transmission of VL.

*L. longispina* is a species found in the Atlantic Forest (Pinto et al. 2008, 2010) and in the peri-urban (Andrade et al. 1997) and urban (Souza et al. 2014) environments. The present study is the first to report its presence in Belo Horizonte, pointing to a possible process of adaptation of other sand fly species of the wild and of the peri-urban areas to the urban environment.

Corroborating other studies, the sand fly captures were higher in the peridomicile compared to the intradomicile areas (Barata et al. 2004, Souza et al. 2004, Dias et al. 2007, 2011, Michalsky et al. 2009, Holcman et al. 2013). Similar data were reported elsewhere in the literature regarding the ratio of males to females (Souza et al. 2004, Dias et al. 2007, de Almeida et al. 2010, Nascimento et al. 2013). The ratio of males to females in the present study was four times higher peridomiciliary (11.6) compared to intradomiciliary (3.4) areas probably because males feed on plant sap and not on blood (Smith et al. 1941, Pessoa & Barreto 1948, Deane et al. 1955). The males form the “mating lek”, i.e. they accompany the females feeding on blood to increase their chance of mating and can be captured more than females (Jarvis & Rutledge 1992). The peridomicile area has sites with animals (hosts), moisture and shady tree trunks, soils with decomposing fruits, leaves and animal feces i.e. full of microenvironments conducive to the reproduction and colonisation by sand flies.

*L. longipalpis* can feed on humans inside their homes and therefore transmit VL at this location (Souza et al. 2004, Barata et al. 2005, Monteiro et al. 2005, Dias et al. 2007, 2011, Michalsky et al. 2009). In the households where the focus of infestation was the peridomicile, the sand flies probably fed mainly on animals such as chickens often raised in those areas since, in nature, they habitually feed on birds (Lainson & Rangel 2005). This was observed in a household located to the south of the studied region (data not shown).

One limitation of the study was the sampling process by convenience adopted by the health agents and the small number of households with a receptivity rating of zero. The convenience sample was used because the study prioritised homes with some degree of receptivity. As a result of the sampling process, another limitation was a presence of one house level two where there was a greater number of captured sand flies (n = 287). This domicile had a large chicken coop surrounding the peridomicile, presence of trees and a lot of organic matter in the soil, and a large yard.

This study is the first to try a system to classify the households regarding their risk of sand fly colonisation. Although the domiciles receptivity levels were not validated, they showed some characteristics that could confirmed the heterogeneity of vector population distribution. The knowledge about the *L. longipalpis* spatial behavior is an important strategy to assist the VL-SCP to combat infestation in its local focus and optimise financial resources invested in vector control. These analyses can contribute to greater effectiveness of VL-SCP in Brazil by directing the vector chemical control and optimise the program’s operational strategies to decrease the sand fly populations and thus reduce the incidence of canine and human VL in urban areas.
